# Identification of (20R)‐protopanaxadiol from *Panax ginseng* as a novel anti‐SARS‐CoV‐2 compound

**DOI:** 10.1002/2211-5463.70307

**Published:** 2026-07-21

**Authors:** Midori Takeda, Natsue Maezono, Rina Uchikoshi, Nobuyuki Kato, Masanori Ikeda

**Affiliations:** ^1^ Division of Biological Information Technology, Joint Research Center for Human Retrovirus Infection Kagoshima University Kagoshima Japan

**Keywords:** antiviral, BAC vector, natural product, replicon, SARS‐CoV‐2

## Abstract

COVID‐19 remains a global health concern, yet current antivirals are restricted to high‐risk patients and face issues of resistance. To establish a safer evaluation system, we constructed a noninfectious SARS‐CoV‐2 replicon using a BAC vector driven by the CMV promoter. Among the tested cell lines, HuH‐7.6c supported the most efficient replication. Replication was inhibited by remdesivir, nirmatrelvir, and molnupiravir, but not by favipiravir or AT‐527. A screening of 373 food‐derived compounds identified (20R)‐protopanaxadiol (PPD), a ginseng metabolite, as a novel inhibitor. Together, this work highlights this BAC‐vectored replicon as a practical platform for antiviral screening and identifies the potential of safe, food‐derived supplements in combating SARS‐CoV‐2.

## Graphical abstract

We established a noninfectious BAC‐based SARS‐CoV‐2 replicon that enables antiviral screening under BSL‐2 conditions. Using this platform, we screened 373 food‐derived compounds and identified (20R)‐protopanaxadiol from Panax ginseng as a novel inhibitor of SARS‐CoV‐2 RNA replication, highlighting the value of safe replicon systems for antiviral discovery.

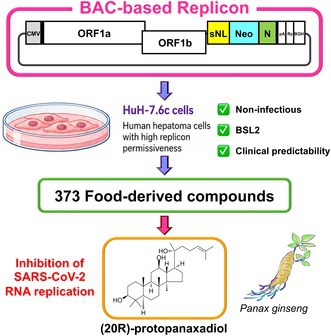

**Abbreviations**
BAC: bacterial artificial chromosome
BSL: biosafety level
COVID‐19: coronavirus disease 2019
EC_50_
: 50% effective concentration
N protein: nucleocapsid protein
PPD: protopanaxadiol
SARS‐CoV‐2: severe acute respiratory syndrome coronavirus‐2

The coronavirus disease 2019 (COVID‐19) pandemic, caused by severe acute respiratory syndrome coronavirus 2 (SARS‐CoV‐2), has had a serious impact on the global health and society of humanity. To reduce the risk of severe illness and mortality, several oral antiviral drugs have been developed and approved for clinical use. These include the RNA‐dependent RNA polymerase inhibitor molnupiravir and the 3CL protease inhibitor nirmatrelvir/ritonavir [1, 2, 3]. However, these drugs are primarily intended for COVID‐19 patients with mild‐to‐moderate symptoms who are not hospitalized but are at high risk of developing severe illness; they are not intended for widespread prescription to general mild cases or asymptomatic cases. Furthermore, the emergence of drug‐resistant strains due to viral mutations has been reported in association with antiviral therapies [4, 5, 6, 7]. Consequently, there is a need for the development of novel antiviral agents that are safer and can be used in a broader population. We considered that antiviral agents with high safety profiles should be developed for preventive or adjunctive use across a broad population.

Food‐derived natural products and plant extracts have long been utilized for the prevention and treatment of infectious diseases, with many reports regarding their antiviral activity. For example, fermented ginseng extract has been reported to improve survival rates and reduce viral loads in mouse models and *in vitro* experiments against influenza virus [8]. Furthermore, ginsenosides were also effective in suppressing infection in *in vitro* and *in vivo* models using influenza A virus (H1N1) [9]. Common dietary nutrients also exhibit antiviral activity; for example, beta‐carotene, vitamin D_2_, and linoleic acid have been reported to inhibit hepatitis C virus (HCV) RNA replication [10]. More recently, *Melissa officinalis* (lemon balm) extract has also shown broad‐spectrum activity against influenza A, SARS‐CoV‐2, and MERS‐CoV *in vitro* [11]. These studies suggest that food‐derived compounds could serve as promising complementary or lead compounds for antiviral drug development. In the context of acute infectious diseases, early suppression of viral replication is critical to prevent symptom exacerbation and the spread of infection. Therefore, continuous intake of safe, food‐derived compounds with the potential to reduce viral load may be beneficial as an adjunctive or preventive strategy, particularly during the early phase of infection.

The study of antiviral compounds against SARS‐CoV‐2, however, is restricted by the requirement of biosafety level 3 (BSL‐3) facilities to handle infectious virus. In many regions, including Europe, Asia, and Australia, the mandatory use of BSL‐3 laboratories has become a major bottleneck in drug screening for many research institutions. Therefore, in this study, we established a replicon system that evaluates only the viral replication process without using infectious viruses. Research on the HCV has significantly advanced the development of anti‐HCV drugs leading to clinical applications using replicon systems [12, 13, 14, 15]. Thus, the replicon system is a powerful experimental system that combines safety and utility, enabling the analysis of viral RNA replication without producing infectious particles. In this study, we established a SARS‐CoV‐2 replicon assay system using synthetic genes constructed from database information, without utilizing the natural SARS‐CoV‐2 virus. The purpose of this study was to use this replicon to identify specific inhibitors of SARS‐CoV‐2 replication from dietary‐derived natural products.

## Materials and methods

### Plasmid construction

The SARS‐CoV‐2 replicon expression plasmid was constructed from 10 artificially synthesized fragments (GenScript, NJ, USA) based on the sequence of the Wuhan‐Hu‐1 strain (GenBank accession no. NC_045512). Each fragment was approximately 2.5 kb in size and contained *Bsa*I restriction enzyme recognition sequences at both ends. After digesting the fragments with *Bsa*I, they were grouped into sets of 3–4 fragments and cloned into the pHSG298 vector (Fragments 1–3, 4–6, 7–10). Then, the fragments were cut out from the vector again with *Bsa*I and introduced into the bacterial artificial chromosome (BAC) vector pSMART BAC v2.0 (#42030‐2, Lucigen, WI, USA). Ligation was performed using the TaKaRa DNA Ligation Kit LONG (#6024, TaKaRa, Shiga, Japan), and transformation was carried out using BAC‐Optimized Replicator v2.0 Electrocompetent Cells (#60210‐2, Lucigen, WI, USA). A polymerase‐defective mutant (SAA; D760A/D761A substitutions in nsp12) was generated using the QuikChange Site‐Directed Mutagenesis Kit (Agilent, Santa Clara, CA, USA) according to the manufacturer's protocol [16].

### Cells lines

Vero cells (derived from African green monkey kidney) were kindly provided by Prof. Eizuru, Kagoshima University. MDCK cells (derived from dog kidney) and BHK‐21 cells (derived from baby hamster kidney) were purchased from the JCRB Cell Bank (MDCK; #JCRB9029, BHK‐21; # JCRB9020). HEK293 cells (RRID:CVCL_0045; derived from human embryonic kidney), Hep3B cells (RRID:CVCL_C8Q9; derived from human hepatocellular carcinoma), and HuH‐7 cells (RRID:CVCL_0336; derived from human hepatocellular carcinoma) were kindly provided by Prof. Kato, Okayama University. HuH‐7.6c cells and RSc cells are subclones derived from HuH‐7 cells [13, 17]. All cell lines were maintained in Dulbecco's Modified Eagle Medium (DMEM) supplemented with 10% fetal bovine serum (FBS) (Thermo Fisher Scientific, MA, USA) at 37°C in a humidified atmosphere containing 5% CO_2_. Human cell lines were obtained from established sources as described above. HuH‐7.6c and HuH‐7.RSc are laboratory‐derived cured subclones established from parental HuH‐7 cells. The parental HuH‐7 cells and both HuH‐7‐derived subclones were characterized by sequencing the full‐length coding region of the human albumin transcript, confirming their human origin. In addition, the HuH‐7‐derived subclones were confirmed to be free of mycoplasma contamination at the time of patent deposition.

### Reagents and antibodies

Medicine Food Homology compound library, remdesivir, favipiravir, and MK‐4482 (EIDD‐1931) were purchased from Selleck Chemicals (TX, USA). PF‐07321332 was purchased from Amadis Chemical (Hangzhou, China), and AT‐527 was purchased from MedChemExpress (NJ, USA). The antibodies used were SARS‐CoV‐2 nucleocapsid (N) protein antibody (#33717, Cell Signaling Technology, MA, USA), β‐actin antibody (#A5441, Sigma‐Aldrich, MO, USA), and mouse IgG (#NA931, GE Healthcare, IL, USA).

### Luciferase assay

The cells (2 × 10^5^ cells·well^−1^) were seeded into a 6‐well plate. After 24 h, 2 μg of the replicon expression vector was transfected using FuGENE® HD Transfection Reagent (Promega, WI, USA). Twenty‐four hours after transfection, cells were detached with trypsin and seeded into a 24‐well plate (2 × 10^4^ cells·well^−1^). The candidate antiviral agents were added 24 h later. The cell culture supernatants were collected 48 h after the addition of the drug. Luciferase activity was measured according to the Nano‐Glo™ Luciferase Assay (Promega) protocol.

### WST‐1 cell proliferation assay

HuH‐7.6c cells (3 × 10^3^ cells) were seeded into 96‐well plates, and candidate antiviral agents were added after 24 h. The cytotoxicity was assessed using the WST‐1 assay according to the Premix WST‐1 Cell Proliferation Assay System protocol (Takara Bio, Shiga, Japan) 48 h after the addition of the drug.

### Western blot analysis

The cells (2 × 10^5^ cells·well^−1^) were seeded into 6‐well plate. After 24 h, 2 μg of the replicon expression vector was introduced into the cells using transfection reagent. After 24 h, the cells were detached with trypsin and seeded into 6‐well plate. Twenty‐four hours after cell seeding, the candidate antiviral drug was added. Forty‐eight hours after addition of drug, the cells were lysed by adding 2× SDS gel loading buffer, and crude protein fractions were recovered by sonication. Preparation of cell lysates, SDS/polyacrylamide gel electrophoresis (SDS/PAGE), and immunoblotting were performed according to previously described methods [18].

### Quantitative RT‐PCR analysis

HuH‐7.6c cells (2 × 10^5^ cells·well^−1^ at 6‐well plate) were cultured for 24 h before the transfection with replicon expression vector. The replicon expression vector (2 μg) was introduced into the cells using transfection reagent. After 24 h, the cells were detached with trypsin and seeded into 6‐well plate. After 24 h, the candidate antiviral drugs were added. After 48 h for treatment, total RNA was extracted using the RNeasy Mini Kit (Qiagen). Each of the RNAs were digested by RQ1 RNase‐free DNase (Promega) at 37°C for 30 min. Then, DNase was inactivated at 65°C for 10 min. Reverse transcription was performed by M‐MLV Reverse Transcriptase (Invitrogen) with Oligo(dT)12–18 primer (Invitrogen). Quantitative PCR was performed using THUNDERBIRD SYBR™ qPCR Mix (TOYOBO) on Applied Biosystems StepOnePlus Real‐Time PCR System. The ORF1a gene was detected using the primers NIID_WH‐1_F509 (5′‐CTCGAACTGCACCTCATGG‐3′) and NIID_WH‐1_R854 (5′‐CAGAAGTTGTTATCGACATAGC‐3′), which were described in a previous report [19]. The GAPDH was detected using the primers hGAPDH (5′‐ GACTCATGACCACAGTCCATGC‐3′) and hGAPDHr (5′‐GAGGAGACCACCTGGTGCTCAG‐3′). The thermal cycling conditions were as follows: 1 min at 95°C for denaturation, 35 cycles of 15 s at 95°C, 15 s at 55°C, 45 s at 72°C. Fluorescence of SYBR Green was detected in each cycle.

### Statistical analysis

Data are presented as mean ± standard deviation (SD) from at least three biological replicates. *P*‐values were determined by Student's *t*‐test. *P < 0.01 was considered statistically significant.

## Results

### Establishment of SARS‐CoV‐2 replicons via artificial gene synthesis

**Fig. 1:**
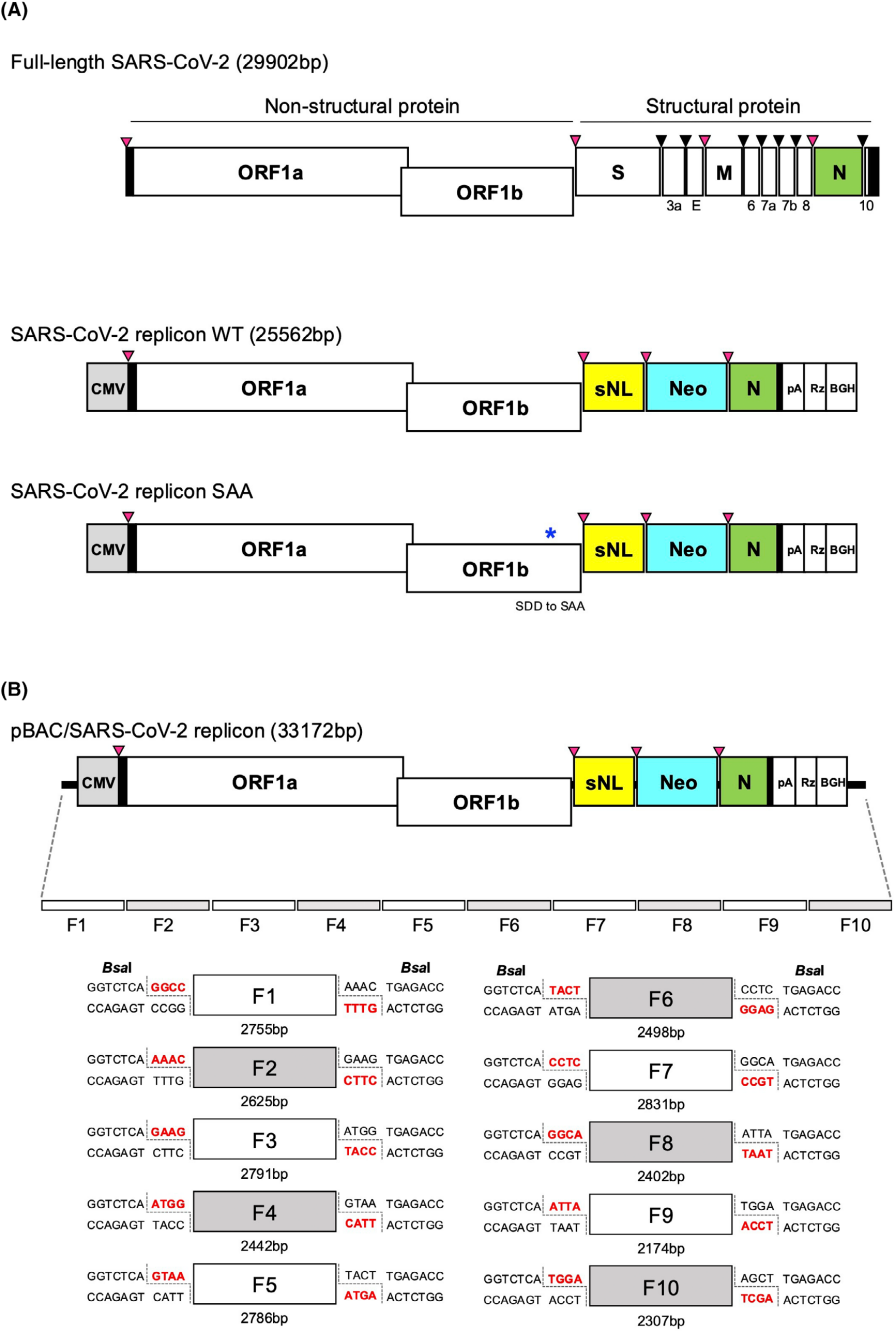
Schematic of the genetic composition of the SARS‐CoV‐2 replicon. (A) The schematics of the SARS‐CoV‐2 full‐length genome (top), wild‐type replicon (WT, middle), and polymerase‐inactive mutant (SAA, bottom). The structural gene region was replaced with secreted Nano‐luciferase gene (sNL), the neomycin resistance gene (Neo), and the nucleocapsid (N). Transcription regulatory sequence in the leader (TRS‐L) and transcription regulatory sequence in the body (TRS‐B) are indicated by pink inverted triangles. SAA: The SDD motif (amino acids 759–761) of the nsp12 protein was replaced with the amino acid sequence of SAA as the negative control. (B) The schematic of the SARS‐CoV‐2 replicon expression vector. The SARS‐CoV‐2 replicon was artificially synthesized by dividing it into 10 fragments of approximately 2.5 kb. Ten synthetic gene fragments were ligated with *Bsa*I and introduced into a bacterial artificial chromosome (BAC) vector.

To assay SARS‐CoV‐2 RNA replication safely, we constructed a SARS‐CoV‐2 replicon assay system. First, we artificially synthesized genes based on the Wuhan‐Hu‐1 strain genome sequence, in which the structural gene regions were replaced with a secreted Nano‐luciferase (sNL) gene and a neomycin resistance gene (Neo), while retaining the N gene. This design enabled quantitative measurement of RNA replication activity without producing viral particles. Furthermore, a mutant replicon with the conserved SDD motif (amino acids 759–761) of the RNA‐dependent RNA polymerase (nsp12) replaced by SAA was created as a negative control to eliminate replication‐independent background signals (Fig. 1A) [20].

To construct the vector, the SARS‐CoV‐2 replicon was artificially synthesized by dividing it into 10 fragments of approximately 2.5 kb each, with *Bsa*I recognition sites added to both ends. These fragments were excised with *Bsa*I and cloned by ligating 3–4 fragments together. Finally, the fragments were excised again and introduced into the BAC vector to complete the replicon expression plasmid (Fig. 1B). The resulting plasmid does not produce infectious particles, allowing for safe handling at BSL‐2 level. It can now serve as a foundation for subsequent antiviral drug screening.

### Comparison of replication efficiency among different cell lines

**Fig. 2:**
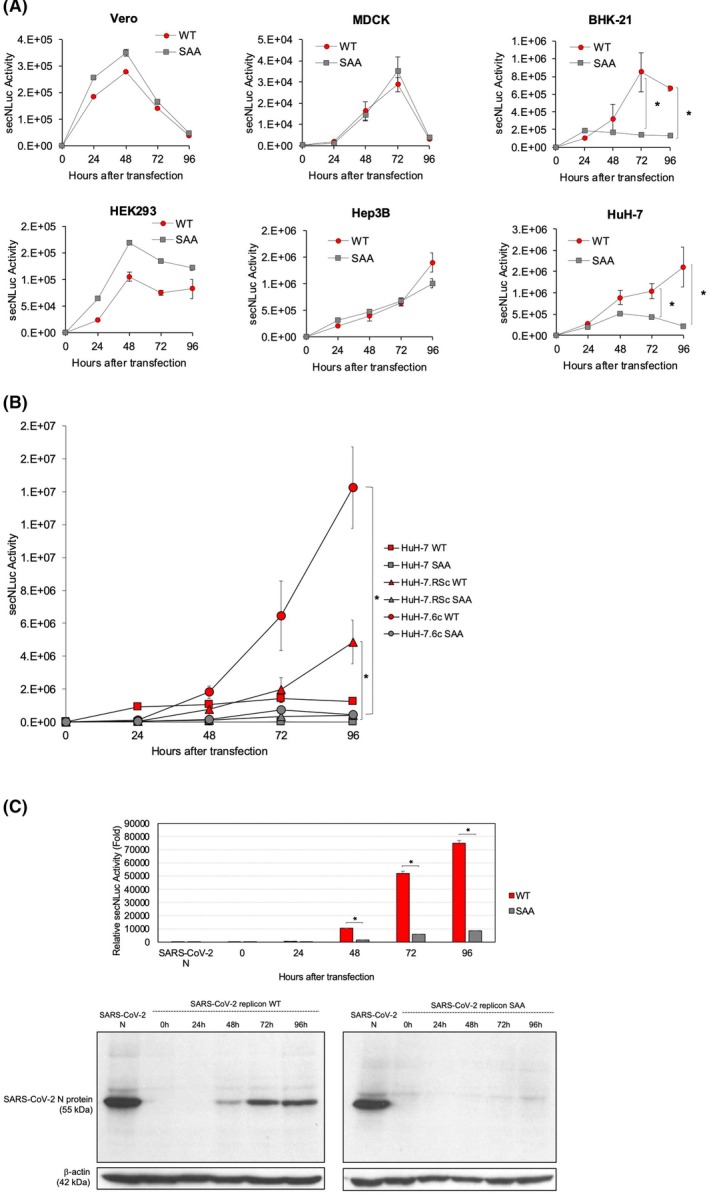
Comparison of SARS‐CoV‐2 replicon replication activity in various cell lines. (A) Wild‐type (WT) or polymerase‐inactivating mutant (SAA) SARS‐CoV‐2 replicons were transfected into Vero, HEK293, MDCK, Hep3B, BHK‐21, and HuH‐7 cells. Luciferase activity was measured 24–96 h post‐transfection. Red circles indicate WT; gray squares indicate SAA. (B) WT or SAA replicons were transfected into HuH‐7, HuH‐7.RSc, and HuH‐7.6c cells. Luciferase activity was measured 24–96 h post‐transfection. (C) After transfection of WT or SAA replicons into HuH‐7.6c cells, luciferase activity was evaluated (top) and nucleocapsid (N) protein expression was detected (bottom) at 24, 48, 72, and 96 h. β‐Actin was used as a control for the amount of protein loaded into each lane. Data are presented as mean ± SD from three biological replicates. Error bars indicate SD. Statistical significance was determined using Student's t‐test. *P < 0.01.

To determine the most permissive host cells, wild‐type (WT) or polymerase inactivation variant (SAA) replicons were first introduced into multiple cell lines, and luciferase activity was measured over time. In Vero and HEK293 cells, the peak luciferase activities were observed 48 h post‐transfection, while in MDCK cells, the peak was observed at 72 h. However, in all cases, SAA showed higher activity than WT, suggesting that the signal was not replication dependent. In Hep3B cells, both WT and SAA showed a time‐dependent increase in activities, but no differences were observed between them. Therefore, SARS‐CoV‐2 RNA replication was not detected in these cells. In contrast, BHK‐21 and HuH‐7 cells exhibited a clear difference in luciferase activity between WT and SAA. Notably, HuH‐7 cells showed time‐dependent and high‐level luciferase activity, suggesting suitability for SARS‐CoV‐2 RNA replication (Fig. 2A). Furthermore, replication efficiency was compared using subclones derived from HuH‐7 cells.

The subclone cells used were HuH‐7.RSc and HuH‐7.6c. These cured cells were generated by eliminating HCV from cells that originally harbored the virus. When WT or SAA replicons were introduced into HuH‐7.RSc and HuH‐7.6c, significant differences were observed between WT and SAA in all subclones, demonstrating higher replication efficiency than the parental strain. Notably, HuH‐7.6c exhibited a marked increase in luciferase activity over time, confirming it as the cell line most efficiently maintaining replication (Fig. 2B). Moreover, evaluation of replicon‐derived protein expression in HuH‐7.6c cells revealed that N protein expression was detected 48 h or later after WT replicon transfection, whereas no expression was detected with the SAA replicon (Fig. 2C). These results suggested that HuH‐7.6c cells are the optimal host cells for SARS‐CoV‐2 replicon analysis, and they were used in subsequent experiments.

### Validation of the replicon using clinically approved antivirals

**Fig. 3:**
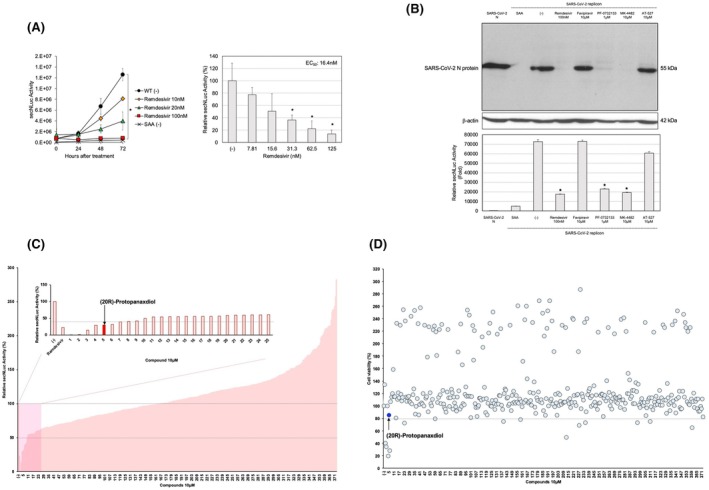
Evaluation of replicon using approved drugs and natural product libraries. (A) HuH‐7.6c cells were transfected with replicon, and remdesivir was added 24 h later. The luciferase activity was evaluated from 24 to 96 h after addition when remdesivir was treated at concentrations of 10, 20, and 100 nm, respectively (left). The luciferase activity was evaluated at 48 h after addition of serially diluted remdesivir, and the 50% effective concentration (EC_50_) value was calculated (right). (B) HuH‐7.6c cells were transfected with the replicon, and approved antiviral drugs were added 24 h later. The nucleocapsid (N) protein expression (top) and the luciferase activity (bottom) were evaluated 48 h after addition of drug. β‐Actin was used as a control for the amount of protein loaded per lane. (C) HuH‐7.6c cells were transfected with replicon. The compound 373 from the food‐derived natural product library was added 24 h post‐transfection. It was diluted to a final concentration of 10 μm. The luciferase activity was measured 48 h later. The untreated group was indicated as (−), and remdesivir (100 nm) was used as a positive control. (D) HuH‐7.6c cells were treated with compound 373. It was diluted to a final concentration of 10 μm. The cell viability was assessed using the WST‐1 cell proliferation assay after 48 h. Data are presented as mean ± SD from three biological replicates. Error bars indicate SD. Statistical significance was determined using Student's t‐test. *P < 0.01.

To evaluate whether this system can reproduce the activity of antiviral drugs, we introduced replicons into HuH‐7.6c cells and validated the utility of the replicon system using approved drugs. Remdesivir, the RNA‐dependent RNA polymerase inhibitor, reduced luciferase activity in a concentration‐dependent and time‐dependent manner between 24 and 96 h, with an EC_50_ of 16.4 nm (Fig. 3A). This confirmed that the replicon system can sensitively reflect the antiviral effect of the drug.

Furthermore, the 3CL‐protease inhibitor PF‐07321332 (nirmatrelvir) and the polymerase inhibitor MK‐4482 (molnupiravir) also markedly suppressed replication. In contrast, favipiravir and AT‐527 had no inhibitory effect. The expression of N protein and ORF1a gene was also consistent with the luciferase assay results, decreasing with remdesivir, PF‐07321332, and MK‐4482, but not with favipiravir and AT‐527 (Fig. 3B; Fig. S1). Drugs with confirmed clinical efficacy were shown to suppress viral RNA replication in the replicon system, whereas favipiravir and AT‐527, whose development was discontinued owing to insufficient clinical efficacy, showed no inhibitory activity. Thus, the replicon system accurately predicted the clinical efficacy profiles of clinically used antivirals.

### Screening of food‐derived natural‐product compounds

Next, we screened for antiviral candidates using a library of food‐derived natural products. After transfection of HuH‐7.6c cells with the replicon, we added 373 compounds and evaluated luciferase activity after 48 h. Luciferase assays identified 10 compounds that suppressed replication by more than 50% compared to the untreated group (Fig. 3C; Table 1). In parallel cell viability assays, six of these compounds were found to maintain survival rates of 80% or higher (Fig. 3D). Among the six compounds, Daurisoline inhibited luciferase activity by 85.9% with the cell viability of 99.7%. Next, (20R)‐protopanaxadiol (PPD) inhibited luciferase activity by 70% with the cell viability of 85.6%. Daurisoline has previously been reported to exhibit antiviral activity against SARS‐CoV‐2 [21]. Therefore, (20R)‐PPD was selected as a promising antiviral candidate compound against SARS‐CoV‐2 (Table 1).

**Table 1:** Identification of antiviral candidate compounds against SARS‐CoV‐2 replication.

	Compound name	Relative secNLuc activity (%)	Cell viability (%)
	(−)	100	100
	Remdesivir	22.8	134.1
1.	Shikonin	0.2	40.0
2.	Periplocin	1.6	34.4
3.	Daurisoline	14.1	99.7
4.	Piperlongumine	29.6	19.3
5.	(20R)‐Protopanaxdiol	30.0	85.6
6.	Protodioscin	31.9	28.1
7.	Dehydrodiisoeugenol	38.9	106.7
8.	Dauricine	40.7	114.3
9.	(S)‐2‐Hydroxysuccinic acid	42.2	119.7
10.	Panaxatriol	49.3	158.6

### Characterization of (20R)‐protopanaxadiol

**Fig. 4:**
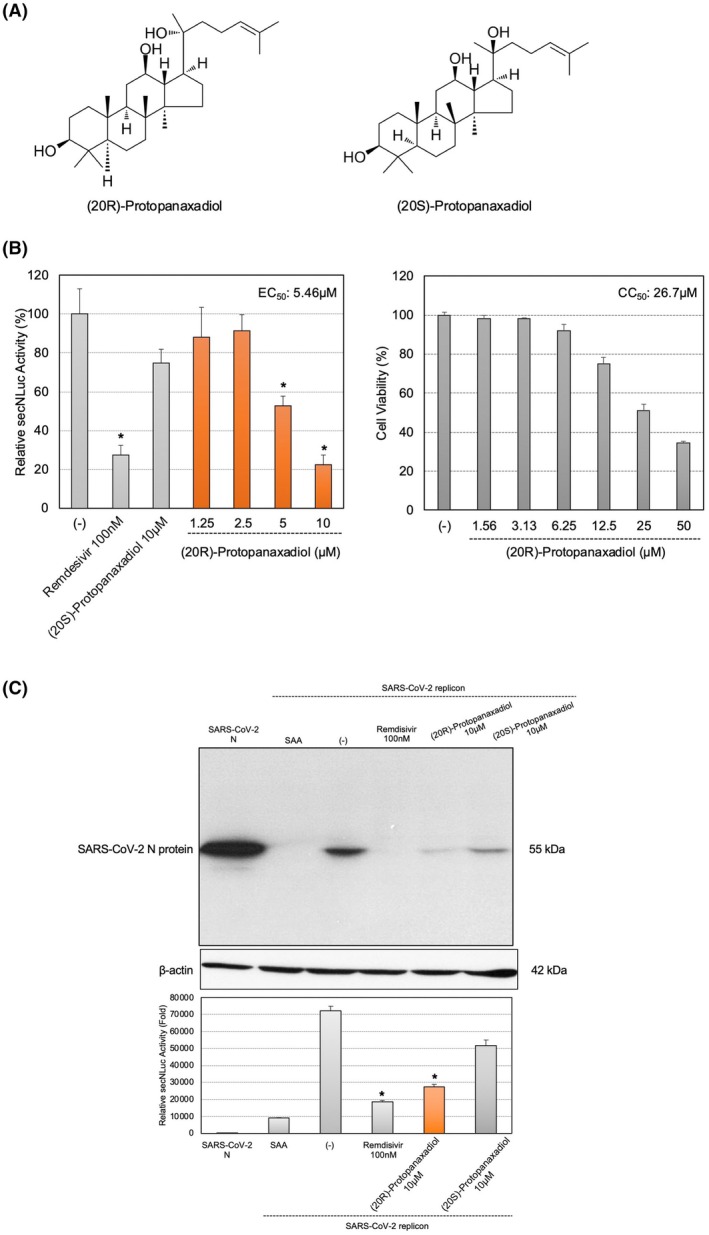
Evaluation of the antiviral activity of (20R)‐protopanaxadiol [(20R)‐PPD]. (A) Chemical structures of (20R)‐protopanaxadiol [(20R)‐PPD] and (20S)‐protopanaxadiol [(20S)‐PPD]. (B) HuH‐7.6c cells were transfected with replicons, and (20R)‐PPD or (20S)‐PPD was added 24 h later. The luciferase activity was measured after 48 h. The 50% effective concentration (EC₅₀) values were calculated for (20R)‐PPD (left). HuH‐7.6c cells were treated with (20R)‐PPD. The cell viability was assessed using the WST‐1 cell proliferation assay after 48 h. The 50% cytotoxic concentration (CC₅₀) values were calculated for (20R)‐PPD (right). (C) HuH‐7.6c cells were transfected with the replicon, and (20R)‐PPD or (20S)‐PPD were added 24 h later. The nucleocapsid (N) protein expression (top) and luciferase activity (bottom) were evaluated 48 h after addition of drug. β‐Actin was used as a control for the amount of protein loaded per lane. Data are presented as mean ± SD from three biological replicates. Error bars indicate SD. Statistical significance was determined using Student's *t*‐test. *P < 0.01.

We examined in detail the antiviral effects of (20R)‐PPD by screening and identifying compounds. The optical isomers, the (20R)‐ and (20S)‐PPD, are shown in Fig. 4A. We investigated whether the structural difference affects antiviral activity.

The (20R)‐PPD inhibited replication in a dose‐dependent manner, exhibiting an EC₅₀ value of 5.46 μm and a CC_50_ value of 26.7 μm (Fig. 4B). Furthermore, (20R)‐PPD significantly repressed the expression of N protein and the ORF1a gene. These results were consistent with the luciferase assay (Fig. 4C; Fig. S2). In contrast, the antiviral activity of (20S)‐PPD was very weak compared to (20R)‐PPD (Fig 4B,C; Fig. S2). Collectively, our findings demonstrate that the BAC‐based SARS‐CoV‐2 replicon represents a robust platform suitable for operation under BSL‐2 conditions. It not only reproduces the antiviral profiles of approved drugs but also enables the efficient identification of novel inhibitors such as the ginseng‐derived metabolite (20R)‐PPD.

## Discussion

In this study, we established a SARS‐CoV‐2 replicon system using the BAC vector that enables quantitative assessment of viral RNA replication under BSL‐2 conditions. A comparison of multiple cell lines revealed that HuH‐7.6c cells exhibited the highest replication efficiency, confirming them as a suitable host cell for antiviral drug evaluation. This replicon system faithfully reproduced the antiviral activity of clinically approved drugs and identified (20R)‐PPD as a novel antiviral candidate compound against SARS‐CoV‐2.

The replicon developed in this study offers several advantages over previously reported systems. Most existing SARS‐CoV‐2 replicons utilize the T7 promoter, which requires *in vitro* transcription followed by the electroporation of RNA into target cells [22, 23, 24, 25]. While these systems are effective for short‐term assays, their luciferase activity peaks 24–48 h after electroporation before declines, thereby limiting the evaluation of compounds that act in the later stages of replication. In contrast, our BAC‐based replicon driven by the CMV promoter can be introduced into cells via simple transfection, eliminating the need for *in vitro* RNA synthesis and electroporation. Furthermore, replication is sustained for at least 4 days, providing a longer time window for compound testing. Several groups have reported replicons using BAC vectors. While these systems improved stability and ease of handling, they lacked polymerase‐deficient controls or verification of protein expression from the replicon [26, 27]. In this study, the use of SAA mutants as replication negative controls enabled clear differentiation between background signals and true replication signals. Additionally, western blot analysis of the N protein confirmed the functionality of the replicon at the protein level. These features enhance the system's reliability, making it suitable for detailed quantitative analysis. In addition to replicon systems, minigenome‐based evaluation systems have also been reported recently [28].

We evaluated multiple cell lines and identified Huh‐7.6c cells as the most efficient host for SARS‐CoV‐2 replicon replication. The observation that subclones derived from HuH‐7 cells exhibit higher replication efficiency than their parental strain has also been reported in HCV replicon studies [29, 30]. Genetic or epigenetic alterations within these subclones likely contribute to a state permissive for RNA virus replication. Our findings suggest that similar host factors may also influence the activity of SARS‐CoV‐2 replicons as well.

Validation of this system using approved drugs confirmed its ability to accurately reflect clinically significant antiviral effects. The RNA‐dependent RNA polymerase inhibitor remdesivir, the 3CL protease inhibitor PF‐07321332 (nirmatrelvir), and the nucleoside analog MK‐4482 (molnupiravir) all exhibited potent, dose‐dependent inhibition of viral replication. In contrast, favipiravir and AT‐527 showed little to no activity, which is consistent with clinical and virological data reporting limited efficacy for these compounds [31, 32, 33]. These results confirm the robustness of the replicon system in predicting pharmacological effects observed in patients and demonstrate its applicability for drug screening. This study has several limitations. The replicon system evaluates viral RNA replication but does not assess viral entry, assembly, or release of infectious particles. Therefore, antiviral candidates identified using this platform should be further validated in infectious virus and *in vivo* models. Nevertheless, the present system reproduced the clinical efficacy profiles of approved antivirals, including the lack of activity of favipiravir and AT‐527, suggesting that host cell selection may substantially influence the clinical relevance of antiviral evaluation systems.

We screened food‐derived compounds using our replicon system and identified (20R)‐PPD, which is a metabolite of *Panax ginseng* ginsenosides, as a novel inhibitor of SARS‐CoV‐2 replication. Korean ginseng (*Panax ginseng*) is widely used in traditional East Asian medicine and modern supplements due to its immunomodulatory, antioxidant, and anti‐inflammatory effects [34, 35]. Ginsenosides and their metabolites are known to exhibit antitumor, anti‐inflammatory, and immunomodulatory effects. Reported mechanisms of action include the induction of caspase‐dependent apoptosis, the reduction of inflammatory cytokine production via suppression of the NF‐κB pathway, and the inhibition of reactive oxygen species production [36, 37]. Previous reviews also support the efficacy of Korean ginseng in respiratory and other infections, suggesting its usefulness as an adjunctive therapy through immunomodulatory and anti‐inflammatory effects [38]. Furthermore, Korean Red Ginseng extract has been shown to reduce inflammation and oxidative stress in a SARS‐CoV‐2 pseudovirus model, demonstrating its potential as an effective antiviral functional food [39]. The results of this study support these findings and further demonstrate that the ginseng metabolite, (20R)‐PPD, inhibits SARS‐CoV‐2 replication. In addition to the discovery of (20R)‐PPD, our findings suggest that food‐derived compounds exhibit potential as a safe and readily available source of antiviral agents. While their efficacy may be lower than that of direct‐acting antivirals, continuous dietary intake or supplementation may contribute to reducing viral load and inflammation during the early stages of infection or following exposure. Therefore, they could be incorporated into preventive or adjunctive treatment strategies. In conclusion, as the history of replicon research demonstrates, noninfectious replication systems have significantly advanced antiviral drug development. The SARS‐CoV‐2 replicon system established in this study is also expected to serve as a powerful platform for screening novel antiviral compounds. As the SARS‐CoV‐2 pandemic continues globally, and in light of the limitations of existing drugs and the growing risk of resistance, the development of antiviral agents derived from safe, daily ingestible supplements, and food components is critically important for future infectious disease control.

In this study, we identified natural compounds that exhibit potent antiviral activity against SARS‐CoV‐2. These findings highlight the potential of safe, food‐derived substances as adjunctive options for COVID‐19 therapy. Further investigations, including *in vivo* and clinical studies, are warranted to validate their efficacy and to support the development of affordable antiviral nutraceuticals for future pandemic preparedness.

## Conflict of interest

The authors declare that they have no conflict of interest.

## Author contributions

MT and MI conceived and designed the study. MT performed the experiments, analyzed and interpreted the data, and prepared the figures. NM and RU contributed to experimental investigation and data acquisition. MT drafted the manuscript. NT and MI reviewed and edited the manuscript. MI supervised the study, administered the project, and acquired funding.

## Supporting information


**Fig. S1.** Evaluation of replicon using approved drugs. HuH‐7.6c cells were transfected with the replicon, and approved drugs were added 24 h later. The quantitative analysis of ORF1a by quantitative reverse transcription PCR (qRT‐PCR) (top) and luciferase activity (bottom) were evaluated 48 h after addition of drug. Glyceraldehyde‐3‐phosphate dehydrogenase (GAPDH) was used as an internal control. Data are presented as mean ± SD from three biological replicates. Error bars indicate SD.
**Fig. S2.** Evaluation of the antiviral activity of (20R)‐protopanaxadiol [(20R)‐PPD]. HuH‐7.6c cells were transfected with replicons, and (20R)‐PPD or (20S)‐protopanaxadiol [(20S)‐PPD] was added 24 h later. The quantitative analysis of ORF1a by quantitative reverse transcription PCR (qRT‐PCR) (top) and luciferase activity (bottom) were evaluated 48 h after addition of drug. Glyceraldehyde‐3‐phosphate dehydrogenase (GAPDH) was used as an internal control. Data are presented as mean ± SD from three biological replicates. Error bars indicate SD.

## Data Availability

The data analyzed in this study are included in the paper. Data will be made available upon request.

## References

[1] Hammond J , Leister‐Tebbe H , Gardner A , Abreu P , Bao W , Wisemandle W , Baniecki M , Hendrick VM , Damle B , Simon‐Campos A *et al*. (2022) Oral nirmatrelvir for high‐risk, nonhospitalized adults with Covid‐19. N Engl J Med 386, 1397–1408. 35172054 10.1056/NEJMoa2118542 PMC8908851

[2] Jayk Bernal A , Gomes da Silva MM , Musungaie DB , Kovalchuk E , Gonzalez A , Delos Reyes V , Martin‐Quiros A , Caraco Y , Williams‐Diaz A , Brown ML *et al*. (2022) Molnupiravir for oral treatment of Covid‐19 in nonhospitalized patients. N Engl J Med 386, 509–520. 34914868 10.1056/NEJMoa2116044 PMC8693688

[3] Hammond J , Fountaine RJ , Yunis C , Fleishaker D , Almas M , Bao W , Wisemandle W , Baniecki ML , Hendrick VM , Kalfov V *et al*. (2024) Nirmatrelvir for vaccinated or unvaccinated adult outpatients with Covid‐19. N Engl J Med 390, 1186–1195. 38598573 10.1056/NEJMoa2309003 PMC11156287

[4] Focosi D , Maggi F , McConnell S and Casadevall A (2022) Very low levels of remdesivir resistance in SARS‐COV‐2 genomes after 18 months of massive usage during the COVID19 pandemic: A GISAID exploratory analysis. Antiviral Res 198, 105247. 35033572 10.1016/j.antiviral.2022.105247 PMC8755559

[5] Gandhi S , Klein J , Robertson AJ , Peña‐Hernández MA , Lin MJ , Roychoudhury P , Lu P , Fournier J , Ferguson D , Mohamed Bakhash SAK *et al*. (2022) De novo emergence of a remdesivir resistance mutation during treatment of persistent SARS‐CoV‐2 infection in an immunocompromised patient: a case report. Nat Commun 13, 1547. 35301314 10.1038/s41467-022-29104-y PMC8930970

[6] Hogan JI , Duerr R , Dimartino D , Marier C , Hochman SE , Mehta S , Wang G and Heguy A (2023) Remdesivir resistance in transplant recipients with persistent coronavirus disease 2019. Clin Infect Dis 76, 342–345. 36156117 10.1093/cid/ciac769 PMC9619446

[7] Heilmann E , Costacurta F , Moghadasi SA , Ye C , Pavan M , Bassani D , Volland A , Ascher C , Weiss AKH , Bante D *et al*. (2023) SARS‐CoV‐2 3CL(pro) mutations selected in a VSV‐based system confer resistance to nirmatrelvir, ensitrelvir, and GC376. Sci Transl Med 15, eabq7360. 36194133 10.1126/scitranslmed.abq7360 PMC9765458

[8] Wang Y , Jung YJ , Kim KH , Kwon Y , Kim YJ , Zhang Z , Kang HS , Wang BZ , Quan FS and Kang SM (2018) Antiviral activity of fermented ginseng extracts against a broad range of influenza viruses. Viruses 10, 471. 30200514 10.3390/v10090471 PMC6164659

[9] Dong W , Farooqui A , Leon AJ and Kelvin DJ (2017) Inhibition of influenza A virus infection by ginsenosides. PLoS One 12, e0171936. 28187149 10.1371/journal.pone.0171936 PMC5302443

[10] Yano M , Ikeda M , Abe K , Dansako H , Ohkoshi S , Aoyagi Y and Kato N (2007) Comprehensive analysis of the effects of ordinary nutrients on hepatitis C virus RNA replication in cell culture. Antimicrob Agents Chemother 51, 2016–2027. 17420205 10.1128/AAC.01426-06 PMC1891399

[11] Alsahafi T , Bouback T , Albeshri A , Alnhhas S , Ali M , Moatasim Y , Kutkat O , Gaballah M , Alfasi F , Mater EH *et al*. (2025) Antiviral potential of *Melissa officinalis* extracts against influenza and emerging coronaviruses. Sci Rep 15, 12118. 40204903 10.1038/s41598-025-96417-5 PMC11982357

[12] Lohmann V , Körner F , Koch J , Herian U , Theilmann L and Bartenschlager R (1999) Replication of subgenomic hepatitis C virus RNAs in a hepatoma cell line. Science 285, 110–113. 10390360 10.1126/science.285.5424.110

[13] Ikeda M , Abe K , Yamada M , Dansako H , Naka K and Kato N (2006) Different anti‐HCV profiles of statins and their potential for combination therapy with interferon. Hepatology 44, 117–125. 16799963 10.1002/hep.21232

[14] Sofia MJ , Bao D , Chang W , Du J , Nagarathnam D , Rachakonda S , Reddy PG , Ross BS , Wang P , Zhang HR *et al*. (2010) Discovery of a β‐d‐2′‐deoxy‐2′‐α‐fluoro‐2′‐β‐C‐methyluridine nucleotide prodrug (PSI‐7977) for the treatment of hepatitis C virus. J Med Chem 53, 7202–7218. 20845908 10.1021/jm100863x

[15] Takeda M , Ikeda M , Mori K , Yano M , Ariumi Y , Dansako H , Wakita T and Kato N (2012) Raloxifene inhibits hepatitis C virus infection and replication. FEBS Open Bio 2, 279–283. 10.1016/j.fob.2012.08.003 PMC3642163 23650611

[16] Toyodome A , Mawatari S , Eguchi H , Takeda M , Kumagai K , Taniyama O , Ijuin S , Sakae H , Tabu K , Oda K *et al*. (2024) Analysis of the susceptibility of refractory hepatitis C virus resistant to nonstructural 5A inhibitors. Sci Rep 14, 16363. 39013947 10.1038/s41598-024-67169-5 PMC11252252

[17] Ariumi Y , Kuroki M , Maki M , Ikeda M , Dansako H , Wakita T and Kato N (2011) The ESCRT system is required for hepatitis C virus production. PLoS One 6, e14517. 21264300 10.1371/journal.pone.0014517 PMC3019154

[18] Li J , Takeda M , Imahatakenaka M and Ikeda M (2024) Identification of dihydroorotate dehydrogenase inhibitor, vidofludimus, as a potent and novel inhibitor for influenza virus. J Med Virol 96, e29372. 38235544 10.1002/jmv.29372

[19] Sharif S , Ikram A , Khurshid A , Salman M , Mehmood N , Arshad Y , Ahmed J , Safdar RM , Rehman L , Mujtaba G *et al*. (2021) Detection of SARs‐CoV‐2 in wastewater using the existing environmental surveillance network: A potential supplementary system for monitoring COVID‐19 transmission. PLoS One 16, e0249568. 34185787 10.1371/journal.pone.0249568 PMC8241060

[20] Gao Y , Yan L , Huang Y , Liu F , Zhao Y , Cao L , Wang T , Sun Q , Ming Z , Zhang L *et al*. (2020) Structure of the RNA‐dependent RNA polymerase from COVID‐19 virus. Science 368, 779–782. 32277040 10.1126/science.abb7498 PMC7164392

[21] Wang P , Luo R , Zhang M , Wang Y , Song T , Tao T , Li Z , Jin L , Zheng H , Chen W *et al*. (2020) A cross‐talk between epithelium and endothelium mediates human alveolar‐capillary injury during SARS‐CoV‐2 infection. Cell Death Dis 11, 1042. 33293527 10.1038/s41419-020-03252-9 PMC7721862

[22] Ricardo‐Lax I , Luna JM , Thao TTN , Le Pen J , Yu Y , Hoffmann HH , Schneider WM , Razooky BS , Fernandez‐Martinez J , Schmidt F *et al*. (2021) Replication and single‐cycle delivery of SARS‐CoV‐2 replicons. Science 374, 1099–1106. 34648371 10.1126/science.abj8430 PMC9007107

[23] He X , Quan S , Xu M , Rodriguez S , Goh SL , Wei J , Fridman A , Koeplinger KA , Carroll SS , Grobler JA *et al*. (2021) Generation of SARS‐CoV‐2 reporter replicon for high‐throughput antiviral screening and testing. Proc Natl Acad Sci USA 118, e2025866118. 33766889 10.1073/pnas.2025866118 PMC8053989

[24] Kotaki T , Xie X , Shi PY and Kameoka M (2021) A PCR amplicon‐based SARS‐CoV‐2 replicon for antiviral evaluation. Sci Rep 11, 2229. 33500537 10.1038/s41598-021-82055-0 PMC7838314

[25] Erdmann M , Wing PAC , Webb I , Kavanagh Williamson M , Jearanaiwitayakul T , Sullivan E , Bazire J , Shytaj IL , McKeating JA , Matthews DA *et al*. (2025) A novel toolkit of SARS‐CoV‐2 sub‐genomic replicons for efficient antiviral screening. Viruses 17, 597. 40431609 10.3390/v17050597 PMC12115450

[26] Liu S , Chou CK , Wu WW , Luan B and Wang TT (2022) Stable cell clones harboring self‐replicating SARS‐CoV‐2 RNAs for drug screen. J Virol 96, e0221621. 35080424 10.1128/jvi.02216-21 PMC8941906

[27] Tanaka T , Saito A , Suzuki T , Miyamoto Y , Takayama K , Okamoto T and Moriishi K (2022) Establishment of a stable SARS‐CoV‐2 replicon system for application in high‐throughput screening. Antiviral Res 199, 105268. 35271914 10.1016/j.antiviral.2022.105268 PMC8900913

[28] Luo Y , Yu F , Zhou M , Liu Y , Xia B , Zhang X , Liu J , Zhang J , Du Y , Li R *et al*. (2021) Engineering a reliable and convenient SARS‐CoV‐2 replicon system for analysis of viral RNA synthesis and screening of antiviral inhibitors. MBio 12, e02754. 33468688 10.1128/mBio.02754-20 PMC7845634

[29] Ikeda M , Abe K , Dansako H , Nakamura T , Naka K and Kato N (2005) Efficient replication of a full‐length hepatitis C virus genome, strain O, in cell culture, and development of a luciferase reporter system. Biochem Biophys Res Commun 329, 1350–1359. 15766575 10.1016/j.bbrc.2005.02.138

[30] Takeda M , Ikeda M , Ariumi Y , Wakita T and Kato N (2012) Development of hepatitis C virus production reporter‐assay systems using two different hepatoma cell lines. J Gen Virol 93, 1422–1431. 22456614 10.1099/vir.0.040725-0

[31] Horga A , Saenz R , Yilmaz G , Simon‐Campos A , Pietropaolo K , Stubbings WJ , Collinson N , Ishak L , Zrinscak B , Belanger B *et al*. (2023) Oral bemnifosbuvir (AT‐527) vs placebo in patients with mild‐to‐moderate COVID‐19 in an outpatient setting (MORNINGSKY). Future Virol doi: 10.2217/fvl-2023-0115 PMC10621114 37928891

[32] Bosaeed M , Alharbi A , Mahmoud E , Alrehily S , Bahlaq M , Gaifer Z , Alturkistani H , Alhagan K , Alshahrani S , Tolbah A *et al*. (2022) Efficacy of favipiravir in adults with mild COVID‐19: a randomized, double‐blind, multicentre, placebo‐controlled clinical trial. Clin Microbiol Infect 28, 602–608. 35026375 10.1016/j.cmi.2021.12.026 PMC8747778

[33] Boffito M , Dolan E , Singh K , Holmes W , Wildum S , Horga A , Pietropaolo K , Zhou XJ , Clinch B , Collinson N *et al*. (2023) A phase 2 randomized trial evaluating the antiviral activity and safety of the direct‐acting antiviral Bemnifosbuvir in ambulatory patients with mild or moderate COVID‐19 (MOONSONG study). Microbiol Spectr 11, e0007723. 37338393 10.1128/spectrum.00077-23 PMC10434175

[34] Park HJ , Kim DH , Park SJ , Kim JM and Ryu JH (2012) Ginseng in traditional herbal prescriptions. J Ginseng Res 36, 225–241. 23717123 10.5142/jgr.2012.36.3.225 PMC3659587

[35] Potenza MA , Montagnani M , Santacroce L , Charitos IA and Bottalico L (2023) Ancient herbal therapy: A brief history of Panax ginseng. J Ginseng Res 47, 359–365. 37252279 10.1016/j.jgr.2022.03.004 PMC10214142

[36] Chen XJ , Zhang XJ , Shui YM , Wan JB and Gao JL (2016) Anticancer activities of protopanaxadiol‐ and protopanaxatriol‐type ginsenosides and their metabolites. Evid Based Complement Alternat Med 2016, 5738694. 27446225 10.1155/2016/5738694 PMC4944051

[37] Zhou Y , Wang Z , Ren S and Li W (2024) Mechanism of action of protopanaxadiol ginsenosides on hepatocellular carcinoma and network pharmacological analysis. Chin Herb Med 16, 548–557. 39606268 10.1016/j.chmed.2024.06.002 PMC11589304

[38] Ratan ZA , Haidere MF , Hong YH , Park SH , Lee JO , Lee J and Cho JY (2021) Pharmacological potential of ginseng and its major component ginsenosides. J Ginseng Res 45, 199–210. 33841000 10.1016/j.jgr.2020.02.004 PMC8020288

[39] Kim HJ , Oh Y , Moon S , Oh J , Kim JH , Lee SH , Hyun SH , Park JH , Ko HK , Hwang J *et al*. (2025) Korean red ginseng relieves the inflammation and oxidative stress induced by pseudo‐typed SARS‐CoV‐2. J Ginseng Res 49, 166–178. 40061481 10.1016/j.jgr.2024.11.004 PMC11889375

