# Supplementary material for: Identification of (20R)‐protopanaxadiol from Panax ginseng as a novel anti‐SARS‐CoV‐2 compound
Source: FEBS Open Bio. 2026 Jul 21:10.1002/2211-5463.70307. Online ahead of print. doi: 10.1002/2211-5463.70307 (PMC13398607; doi:10.1002/2211-5463.70307)
Supplement: Supplementary file 1 — Fig. S1. Evaluation of replicon using approved drugs. HuH‐7.6c cells were transfected with the replicon, and approved drugs were added 24 h later. The quantitative analysis of ORF1a by quantitative reverse transcription PCR (qRT‐PCR) (top) and luciferase activity (bottom) were evaluated 48 h after addition of drug. Glyceraldehyde‐3‐phosphate dehydrogenase (GAPDH) was used as an internal control. Data are presented as mean ± SD from three biological replicates. Error bars indicate SD. Fig. S2. Evaluation of the antiviral activity of (20R)‐protopanaxadiol [(20R)‐PPD]. HuH‐7.6c cells were transfected with replicons, and (20R)‐PPD or (20S)‐protopanaxadiol [(20S)‐PPD] was added 24 h later. The quantitative analysis of ORF1a by quantitative reverse transcription PCR (qRT‐PCR) (top) and luciferase activity (bottom) were evaluated 48 h after addition of drug. Glyceraldehyde‐3‐phosphate dehydrogenase (GAPDH) was used as an internal control. Data are presented as mean ± SD from three biological replicates. Error bars indicate SD. [file FEB4-9999-0-s001.docx]

**Supporting Information**

**Identification of (20R)-Protopanaxadiol from *Panax ginseng* as a novel anti-SARS-CoV-2 compound using a BAC-vectored replicon system**

Midori Takeda^1^*, Natsue Maezono^1^, Rina Uchikoshi^1^, Nobuyuki Kato^1^, Masanori Ikeda^1^*

^1^ Division of Biological Information Technology, Joint Research Center for Human Retrovirus Infection, Kagoshima University, Kagoshima, Japan

* **Correspondence:** Division of Biological Information Technology, Joint Research Center for Human Retrovirus Infection, Kagoshima University, 8-35-1 Sakuragaoka,

Kagoshima, Japan.

FAX: +81-99-275-5937

Tal: +81-99-275-5935

*E-mail address*: [maikeda@m3.kufm.kagoshima-u.ac.jp](mailto:maikeda@m3.kufm.kagoshima-u.ac.jp) (M. Ikeda).

*E-mail address*: takemi02@m3.kufm.kagoshima-u.ac.jp (M. Takeda).

**Fig. S1.** Evaluation of replicon using approved drugs.

HuH-7.6c cells were transfected with the replicon, and approved drugs were added 24 hours later. The quantitative analysis of ORF1a by quantitative reverse transcription PCR (qRT-PCR) (top) and luciferase activity (bottom) were evaluated 48 hours after addition of drug. Glyceraldehyde-3-phosphate dehydrogenase (GAPDH) was used as an internal control. Data are presented as mean ± SD from three biological replicates. Error bars indicate SD.

**Fig. S2.** Evaluation of the antiviral activity of (20R)-protopanaxadiol [(20R)-PPD].

HuH-7.6c cells were transfected with replicons, and (20R)-PPD or (20S)-protopanaxadiol [(20S)-PPD] was added 24 hours later. The quantitative analysis of ORF1a by quantitative reverse transcription PCR (qRT-PCR) (top) and luciferase activity (bottom) were evaluated 48 hours after addition of drug. Glyceraldehyde-3-phosphate dehydrogenase (GAPDH) was used as an internal control. Data are presented as mean ± SD from three biological replicates. Error bars indicate SD.
